# Near-Infrared II Imaging and Surgical Anatomical Structure Transparentization: An Emerging Framework for Precision Surgery

**DOI:** 10.34133/research.1371

**Published:** 2026-07-27

**Authors:** Hui Lin, Xiaoxiao Fan, Xiaolong Liu

**Affiliations:** ^1^ Department of General Surgery, Sir Run Run Shaw Hospital, School of Medicine, Zhejiang University, Hangzhou 310016, China.; ^2^ College of Biomedical Engineering and Instrument Science, Zhejiang University, Hangzhou 310027, China.; ^3^ Zhejiang Key Laboratory of Intelligent Medical Decision Support, Taizhou Institute of Zhejiang University, Taizhou 318000, China.

## Abstract

Precision surgery in soft tissues is currently hindered by the lack of real-time, depth-resolved visualization. Conventional navigation relies on preoperative images that fail to account for intraoperative deformation, while existing intraoperative tools often lack intuitive spatial correspondence. Here, we propose Surgical Anatomical Structure Transparentization (SAST), an emerging framework anchored by second near-infrared fluorescence imaging. Unlike augmented reality, which merely displays static preoperative models, SAST represents a sensing paradigm that utilizes direct photon scattering suppression to visualize deep anatomy in situ. We delineate the critical technological trajectory—from monocular 2-dimensional fluorescence to 3-dimensional volumetric perception via stereoscopic vision and computational imaging—required to bridge the dimensional gap. By transforming navigation from “referencing a map” to “seeing through tissue”, SAST promises to redefine surgical perception through continuous, real-time anatomical transparency.

## Introduction

Achieving precision surgery demands that surgeons continuously and accurately recognize critical anatomical structures amidst complex, dynamic intraoperative conditions. In this Perspective, we propose an emerging clinical framework driven by recent advances in optical imaging.

Current surgical navigation systems generally fall into 2 categories: those based on preoperative image reconstruction and those relying on real-time intraoperative acquisition. While the former suits rigid tissues (e.g., bone), it fundamentally fails in soft tissue surgery due to deformation. The latter, represented by intraoperative ultrasound, computed tomography, magnetic resonance imaging, and fluorescence imaging, provides valuable structural or functional information but often lacks intuitive spatial correspondence with the surgical field. This forces surgeons to mentally map disparate images onto the patient’s anatomy, increasing cognitive load [1]. Among these, fluorescence imaging offers dynamic, continuous visualization seamlessly integrated into the surgical workflow; however, current clinical systems utilizing the visible or first near-infrared (NIR-I) windows are hampered by tissue autofluorescence and limited penetration depth [2]. These limitations highlight a fundamental gap: Current navigation systems are not fully integrated into the surgeon’s natural perception. Next-generation navigation necessitates an intraoperative, real-time system capable of deep anatomical visualization.

## NIR-II Fluorescence: The Physical Foundation

The second near-infrared (NIR-II, 900 to 1,880 nm) window offers a compelling solution. By minimizing photon scattering and tissue autofluorescence [3], NIR-II enables superior deep-tissue visualization compared to the NIR-I window. Empowered by advanced sensors and novel probes, NIR-II technology uniquely achieves centimeter-scale penetration (typically 1 to 3 cm in biological tissues) with micron-to-millimeter scale spatial resolution (approx. 50 to 100 μm in ideal subcutaneous models) [4]. Crucially, the clinical significance of these metrics lies in their dual-scale utility: Centimeter-scale penetration allows the identification of deep-seated targets without high-risk mechanical dissection, while submillimeter resolution preserves adjacent delicate structures to ensure negative margins. This unique performance benchmark makes it one of the few modalities capable of satisfying the dual requirements of deep-tissue visibility and real-time video-rate feedback. Consequently, its expanding application in hepatobiliary and orthopedic surgeries highlights substantial clinical potential, while firmly validating its translational stability and safety [4–6].

Technologically, NIR-II imaging integrates a continuous-wave laser, long-pass filters, and an InGaAs camera to capture deep-tissue photons, currently leveraging the tail emission (900 to 1,300 nm) of clinical indocyanine green. Next-generation probes further optimize quantum yields via chromophore engineering and surface modification. This sensing framework can adapt to various diseases across 3 dimensions: tailoring administration routes (systemic vs. local mapping), exploiting inherent pharmacokinetic clearance for real-time angiography, or conjugating chromophores with disease-specific ligands such as RGD peptides for targeted malignancy definition.

## Defining SAST: An Emerging Framework

**Fig. 1.**
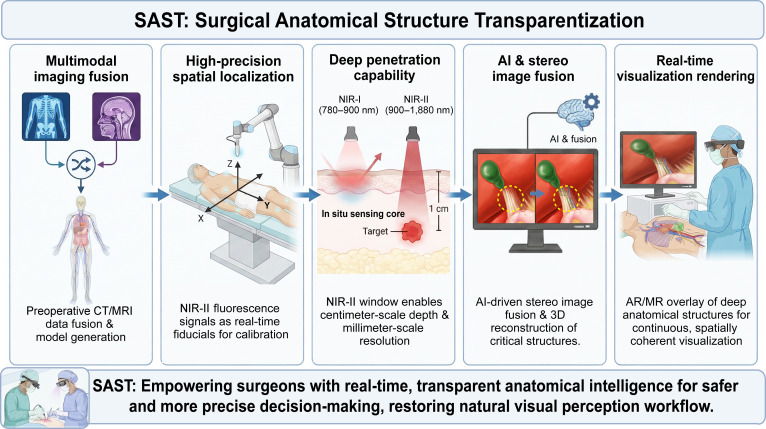
Schematic illustration of the Surgical Anatomical Structure Transparentization (SAST) workflow. This 5-step paradigm integrates multimodal preoperative imaging fusion, high-precision spatial localization, and the deep-tissue penetration capability of second near-infrared (NIR-II) technology. It utilizes artificial intelligence (AI)-driven stereo image fusion to reconstruct and identify critical structures, ultimately providing the surgeon with a real-time augmented reality (AR)/mixed reality (MR) visualization overlay for precise surgical guidance. CT, computed tomography; MRI, magnetic resonance imaging.

Based on these advances, we propose Surgical Anatomical Structure Transparentization (SAST). The schematic illustration of the SAST workflow is shown in Fig. 1. SAST represents an emerging computational and imaging framework that integrates real-time deep-tissue molecular imaging (primarily NIR-II) with dynamically updated 3-dimensional (3D) anatomical models to visually project subsurface structures continuously onto the surgical field. By leveraging intelligent algorithms, this workflow achieves continuous, spatially coherent visualization of deep structures directly within the surgeon’s natural field of view. Ultimately, SAST aims to render the surgical field “transparent”, not to replace clinical judgment but to expand perceptual bandwidth by revealing critical anatomy beyond the reach of natural vision. Compared to current navigation modalities, SAST addresses critical operational bottlenecks [1]. While intraoperative computed tomography/magnetic resonance imaging lacks video-rate feedback and restricts surgical access, and ultrasound demands constant manual manipulation, conventional NIR-I fluorescence suffers from superficial penetration (<5 mm). To highlight how SAST overcomes these technical trade-offs, a comprehensive comparison is provided in Table S1.

## Beyond Display: SAST as an Integrated Sensing-Visualization Framework

It is essential to clarify that SAST is not a substitute for augmented reality (AR), but rather an integrated framework that encompasses AR while extending beyond simple visualization. Current surgical AR systems primarily function as display interfaces that overlay static preoperative data, which inherently fail to account for dynamic soft-tissue deformation in real time. Taking hepatobiliary surgery as a prime example, the liver morphs substantially upon surgical mobilization and retraction, rendering static preoperative 3D vascular roadmaps mismatched with the actual field. SAST redefines this workflow as a closed-loop sensing paradigm. By capturing high-penetration NIR-II photons emerging through centimeters of overlying liver tissue, the system treats these deep optical signals as live, in situ physical landmarks that reflect the actual, real-time positions of subsurface structures. A physics-informed computational inverse algorithm utilizes these live fluorescence constraints to mathematically warp and instantly correct the deformed preoperative 3D roadmap (detailed workflow specifics are presented in the Supplementary Materials). Consequently, SAST shifts the surgical focus from “displaying a historical map” to “sensing and rendering anatomical reality” de novo.

## The Dimensional Challenge: Bridging 2D Signals and 3D Perception

A critical hurdle for SAST is the dimensionality of optical signals. Intrinsic fluorescence provides 2D planar projections, lacking the Z-axis depth required for true volumetric “transparentization”. Solving this ill-posed inverse problem—recovering 3D morphology from scattering-blurred 2D signals—requires converging advanced photonics with computational imaging. To achieve this, a mature SAST system must integrate both hardware and algorithmic pathways. On the hardware front, stereoscopic NIR-II imaging recovers initial depth topology via binocular parallax, while multimodal fusion (e.g., integrating ultrasound or light detection and ranging) provides essential structural boundary constraints. On the algorithmic front, these hardware inputs must be processed through advanced computational depth retrieval. This hinges on a physics-informed deep learning architecture designed to overcome the deterministic limits of light scattering. Specifically, Monte Carlo-based light propagation models are employed to simulate tissue-specific scattering phase functions. These physical priors are then embedded into deep learning-based deconvolution algorithms, which computationally “re-focus” the diffused signals by learning the inverse mapping of the point spread function at varying depths. By synergizing multidimensional optical acquisition with this physics-informed inverse reconstruction, the SAST framework effectively transforms flat, blurred fluorescence into a spatially coherent, 3D volumetric representation, ultimately providing the surgeon with a high-fidelity, transparent view of the deep operative field.

## Outlook

Realizing SAST faces challenges, including camera miniaturization, probe functionalization [7], and algorithm optimization. However, the trajectory is clear: Surgery is transitioning from static, image-based planning toward real-time, perception-augmented execution. NIR-II imaging is the cornerstone of this transition. By anchoring the SAST framework, we move closer to a future where surgeons possess continuous, transparent anatomical intelligence, fundamentally enhancing the safety and precision of operative care.

## Supplementary Materials

Supplementary 1 Table S1 Notes S1 to S5

## References

[1] Fan X, Liu X, Xia Q, Chen G, Cheng J, Shi Z, Fang Y, Khadaroo PA, Qian J, Lin H. Advanced image-guidance and surgical-navigation techniques for real-time visualized surgery. Adv Sci. 2025;12(41): Article e09294. 10.1002/advs.202509294 PMC12591151 40985285

[2] Mieog JSD, Achterberg FB, Zlitni A, Hutteman M, Burggraaf J, Swijnenburg R-J, Gioux S, Vahrmeijer A. Fundamentals and developments in fluorescence-guided cancer surgery. Nat Rev Clin Oncol. 2022;19(1):9–22. 34493858 10.1038/s41571-021-00548-3

[3] Feng Z, Tang T, Wu T, Yu X, Zhang Y, Wang M, Zheng J, Ying Y, Chen S, Zhou J, et al. Perfecting and extending the near-infrared imaging window. Light Sci Appl. 2021;10(1):197. 34561416 10.1038/s41377-021-00628-0 PMC8463572

[4] Hu Z, Fang C, Li B, Zhang Z, Cao C, Cai M, Su S, Sun X, Shi X, Li C, et al. First-in-human liver-tumour surgery guided by multispectral fluorescence imaging in the visible and near-infrared-I/II windows. Nat Biomed Eng. 2020;4(3):259–271. 31873212 10.1038/s41551-019-0494-0

[5] Zhang Z, Du Y, Shi X, Wang K, Qu Q, Liang Q, Ma X, He K, Chi C, Tang J, et al. NIR-II light in clinical oncology: Opportunities and challenges. Nat Rev Clin Oncol. 2024;21(6):449–467. 38693335 10.1038/s41571-024-00892-0

[6] Fan X, Yang J, Ni H, Xia Q, Liu X, Wu T, Li L, Prasad PN, Liu C, Lin H, et al. Initial experience of NIR-II fluorescence imaging-guided surgery in foot and ankle surgery. Engineering. 2024;40(9):20–29.

[7] Hu D, Zha M, Zheng H, Gao D, Sheng Z. Recent advances in Indocyanine green-based probes for second near-infrared fluorescence imaging and therapy. Research. 2025;8:0583. 39830366 10.34133/research.0583 PMC11739436

