# Supplementary material for: Near-Infrared II Imaging and Surgical Anatomical Structure Transparentization: An Emerging Framework for Precision Surgery
Source: Research (Wash D C). 2026 Jul 27;9:1371. doi: 10.34133/research.1371 (PMC13402721; doi:10.34133/research.1371)
Supplement: Supplementary 1 — Table S1 Notes S1 to S5 [file research.1371.f1.docx]

**Near-Infrared II Imaging and** **Surgical Anatomical Structure Transparentization (SAST): An Emerging Framework for Precision Surgery**

Hui Lin ^a,b,c,#,*^, Xiaoxiao Fan ^a,#^, Xiaolong Liu ^a^

**Supplementary Table 1. Comparison of SAST with Conventional Intraoperative Imaging Modalities.**

| **Feature** | **CT/ MRI** | **iUS** | **NIR-I Fluorescence** | **SAST (NIR-II Fluorescence)** |
| --- | --- | --- | --- | --- |
| **Penetration** | High (> 10 cm) | High | < 5 mm | 1–3 cm |
| **Resolution** | Low/Moderate | Low/Moderate | High | High (Sub-mm) |
| **Real-time** | No  (Static) | Yes (Intermittent) | Yes  (Continuous) | Yes  (Continuous) |
| **Specificity** | Structural | Structural | Molecular (Targeted) | Molecular  (Targeted) |
| **Spatial Correspondence** | Poor (Mental mapping) | Moderate (Off-screen) | High  (Surface only) | Superior  (In-situ Transparency) |

**Supplementary Note: Implementation of the SAST Framework in a Generalized Hepatobiliary Scenario**

To demonstrate the practical workflow of the Surgical Anatomical Structure Transparentization (SAST) framework, we delineate its application in a generalized clinical scenario: hepatobiliary surgery involving deep-seated lesions and intricate vascular structures prone to intraoperative deformation.

**1. Preoperative Multimodal Imaging Fusion and Roadmap Generation**

The workflow initiates with multimodal imaging fusion. Preoperative contrast-enhanced computed tomography (CT) or magnetic resonance imaging (MRI) datasets are computationally processed to reconstruct high-fidelity, 3D digital roadmaps. These digital models delineate the patient's intricate internal hepatic vascular architecture and precisely outline the spatial boundaries of deep-seated target lesions.

**2. Intraoperative Spatial Localization and Initial Alignment**

Subsequently, high-precision spatial localization is executed within the operative theater. Utilizing an optical registration system, the preoperative 3D structural roadmap is initially co-registered with the anatomically exposed liver surface, establishing a baseline spatial coordinate alignment.

**3. The Bottleneck of Soft-Tissue Elastic Deformation**

A critical challenge in navigating parenchymal organs is dynamic, soft-tissue elastic deformation. During surgical mobilization, retraction, and parenchymal transection, the liver undergoes substantial morphological shifting. Consequently, the static preoperative 3D digital roadmaps inevitably become misaligned with the actual, dynamic intraoperative surgical field, rendering conventional static overlays unreliable.

**4. Deep-Penetration NIR-II Acquisition and Computational Inverse Correction**

To resolve this compliance mismatch, the SAST framework leverages deep-penetration acquisition coupled with computational image fusion: Probe Kinematics: Clinically approved Indocyanine Green (ICG) is administered to achieve targeted accumulation within the lesions or specific anatomical segments. Photon Capture: Upon surgical exposure of the liver, an overhead indium gallium arsenide (InGaAs) camera system captures high-penetration NIR-II photons emerging through centimeters of overlying healthy parenchyma. Algorithmic Correction: Because these deep optical signals capture the actual, real-time positions of sub-surface structures, they serve as live, in-situ physical constraints. A physics-informed computational inverse algorithm utilizes these intraoperative fluorescence profiles to quantify tissue displacement, mathematically warping and instantly correcting the deformed preoperative 3D roadmap to match the live anatomy.

**5. Real-Time Visualization Rendering and Spatial Coherence Maintenance**

During the final visualization rendering phase, the dynamically corrected, deformation-resilient roadmap is projected as a co-registered augmented reality (AR) overlay onto wearable smart glasses or an overhead surgical display. By utilizing continuous intraoperative NIR-II signals as live in-situ landmarks to constantly compensate for soft-tissue compliance, the SAST framework maintains absolute spatial coherence, enabling the precise, real-time visualization of deep-seated anatomy throughout the hepatobiliary procedure.
